# Including malnourished siblings in treatment improves nutritional outcomes for children with sickle cell anemia in Northern Nigeria: Results from a feasibility trial

**DOI:** 10.1016/j.nutres.2025.10.006

**Published:** 2025-10-18

**Authors:** Hassan Adam Murtala, Shehu U. Abdullahi, Safiya Gambo, Halima Kabir, Khadija A. Shamsu, Garba Gwarzo, Sari A. Acra, Virginia A. Stallings, Mark Rodeghier, Michael R. DeBaun, Lauren J. Klein

**Affiliations:** a Department of Pediatrics, Murtala Muhammad Specialist Hospital, Kano, Nigeria; b Department of Pediatrics, Bayero University/Aminu Kano Teaching Hospital, Kano, Nigeria; c Department of Pediatrics, D. Brent Polk Division of Pediatric Gastroenterology, Hepatology, and Nutrition at Monroe Carell Jr. Children’s Hospital at Vanderbilt, Nashville, TN, USA; d Division of Gastroenterology, Hepatology, and Nutrition, Department of Pediatrics, The Children’s Hospital of Philadelphia and University of Pennsylvania, Philadelphia, PA, USA; e Rodeghier Consultants, Chicago, IL, USA; f Department of Pediatrics, Vanderbilt-Meharry Center of Excellence in Sickle Cell Disease, Vanderbilt University Medical Center, Nashville, TN, USA; g Vanderbilt Institute for Global Health, Vanderbilt University Medical Center, Nashville, TN, USA

**Keywords:** Sickle cell anemia, Siblings, Body mass index, Malnutrition, Child nutrition disorders

## Abstract

Best treatment approaches for malnutrition in children with sickle cell anemia (SCA) remain underexplored. We hypothesized that (1) children with SCA (CwSCA) enrolled in a malnutrition trial alongside their non-SCA siblings would experience greater nutritional improvements than those without an enrolled sibling and (2) enrolled malnourished siblings without SCA would have higher baseline nutritional status and greater improvements in nutritional status than CwSCA. We tested these hypotheses as part of a randomized controlled feasibility trial at 2 medical centers in northern Nigeria, a low-resource setting with a significant burden of malnutrition and SCA. Participants included 108 CwSCA (5-12 years) with severe malnutrition (body mass index (BMI) z-score <−3.0), 21 of whom had an enrolled sibling (Sibling) with severe malnutrition but without SCA (5-12 years, n = 22). All participants received daily ready-to-use therapeutic food (RUTF) for 12 weeks. CwSCA with a Sibling had a higher mean BMI z-score change than CwSCA without a Sibling (0.8 vs 0.4, *P* = .003). The mean baseline BMI z-scores for the CwSCA (−3.7) were comparable to those of their Siblings (−3.6; *P* = .47). Improvement in BMI z-score was similar between CwSCA and their malnourished siblings without SCA. In conclusion, our findings suggest that including malnourished siblings in nutritional interventions enhances outcomes for CwSCA. We postulate that the additional calories delivered by co-treating siblings reduce intrahousehold competition for RUTF, thereby allowing CwSCA to consume a greater share of the therapeutic food. This trial was registered at clinicaltrials.gov (NCT03634488).

## 1. Introduction

Malnutrition is one of the most critical global health challenges, driving millions of preventable deaths each year and exacerbating the burden of disease worldwide [1–3]. Despite the public health impact, the nutritional challenges for children in middle childhood (ages 5 to 12 years) have often been overlooked, with the global focus primarily on children under 5 [4,5]. Community-based management of acute malnutrition with ready-to-use therapeutic food (RUTF) has increased treatment coverage and improved outcomes for severe acute malnutrition in children under 5 [6]. However, there are no evidence-based guidelines for treating malnutrition in children over 5.

Malnourished children with chronic diseases that affect growth, such as sickle cell anemia (SCA), are of particular concern. Children with SCA (CwSCA) have high nutrient demands and energy expenditures and are at a higher risk of malnutrition [7,8]. Notably, increasing age is a clinically relevant risk factor for worse nutritional status in CwSCA in low- and high-income countries [9]. In Nigeria, a low-income country that accounts for over one-third of the global burden of SCA [10], poor nutrition is substantial across the life course [11]. Importantly, older children with SCA living in Nigeria who are underweight are at increased risk for death [12]. However, there are no WHO guidelines for managing malnutrition in children ≥ 5 years of age. To address this absence of evidence-based guidelines, we conducted a randomized controlled feasibility trial in Nigeria to evaluate the management of severe acute malnutrition in CwSCA aged 5-12 years old (NCT03634488) [13]. Our results demonstrated the feasibility and safety of outpatient management of severe acute malnutrition in CwSCA aged 512 years old [13].

As part of this study, siblings aged 5-12 years old without SCA and with severe acute malnutrition were enrolled, as we expected siblings to share the RUTF. Furthermore, there is emerging evidence that nutrition interventions can positively affect siblings [14]. In a sub-study of our severe acute malnutrition feasibility trial [13], we tested the hypotheses that (1) CwSCA with a sibling participating in the malnutrition trial would have a greater improvement in nutritional status than a CwSCA without a sibling enrolled; and (2) enrolled siblings would have a higher nutritional status at baseline and experience greater improvement in nutritional status than CwSCA, despite similar nutrition support.

## 2. Methods and materials

### 2.1. Study design and participants

The randomized controlled feasibility trial for managing severe acute malnutrition (NCT03634488) was conducted at Aminu Kano Teaching Hospital (AKTH) and Murtala Muhammad Specialist Hospital (MMSH), in Kano, northern Nigeria, West Africa [13]. The institutional review boards of AKTH (NHREC/28/01/2020/AKTH/EC/3306), MMSH (NHREC/17/03/2018), and Vanderbilt University Medical Center (190439) granted ethical approval for the study.

Our previous report details the methods and protocols utilized in this study [13]. In brief, CwSCA aged 5-12 years old with laboratory-confirmed SCA (HbSS or HbS-beta^0^ thalassemia) and uncomplicated severe acute malnutrition, as determined by a body mass index (BMI) z-score <−3.0 according to the World Health Organization growth reference, were enrolled [15]. Siblings of CwSCA who did not have SCA were invited for screening and enrollment if they had uncomplicated severe acute malnutrition with a BMI z-score of less than −3.0. Enrolled siblings who did not have SCA will be referred to as “Siblings.”

### 2.2. Study procedures

Enrolled children (CwSCA and Siblings) aged 5.00-8.99 years received one daily sachet of RUTF (500 calories) each, and those aged 9.00-12.99 years received 2 daily sachets of RUTF (1000 calories) each. The CwSCA were randomized, half receiving moderate-dose hydroxyurea (20 mg/kg/d). All enrolled children (CwSCA and Siblings) were evaluated in the first week to assess for refeeding syndrome, followed by scheduled study visits every 4 weeks for 12 weeks.

### 2.3. Statistical analyses

**Fig. 1 –:**
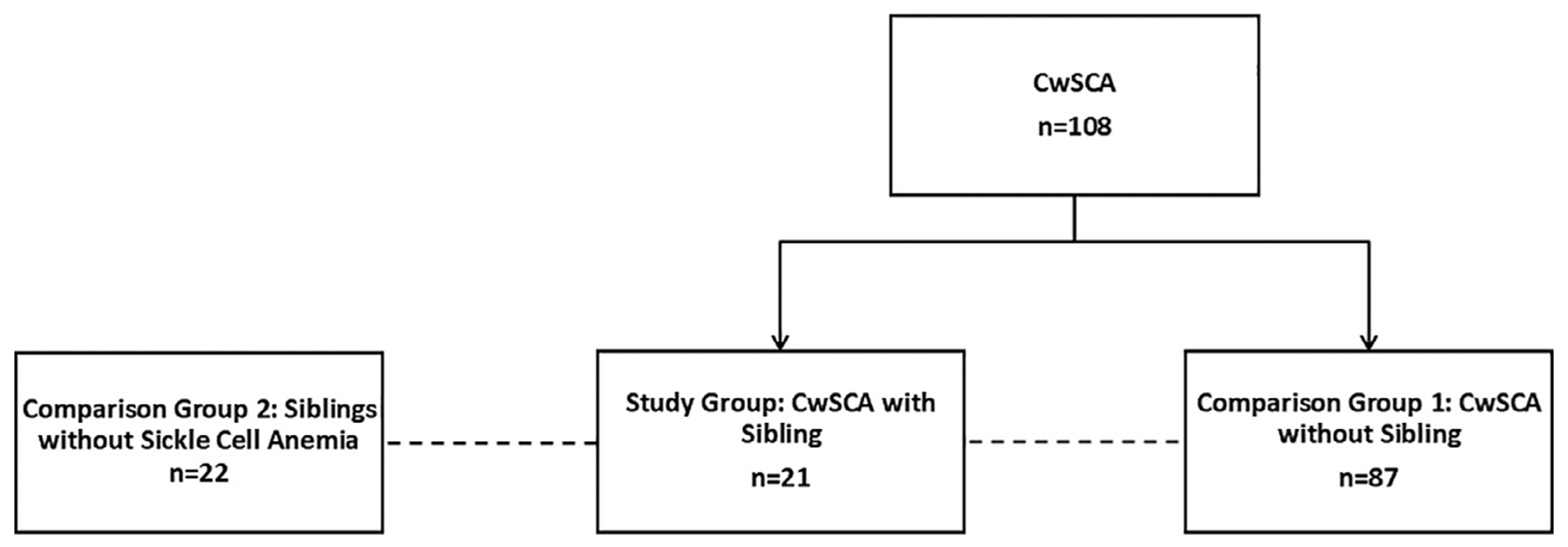
Study diagram for children with severe acute malnutrition (body mass index z-score <−3.0), comparing children with sickle cell anemia with an enrolled sibling without sickle cell anemia (n = 21), children with sickle cell anemia without an enrolled sibling (n = 87), and siblings (n = 22). Abbreviations: BMI = Body Mass Index; CwSCA = Children with sickle cell anemia

Data were systematically collected and managed using Research Electronic Data Capture (REDCap) [16]. For this sub-study, the study group consisted of CwSCA with an enrolled sibling (CwSCA with Sibling). The comparison groups, each compared separately with the study group, were (1) CwSCA without an enrolled sibling (CwSCA without Sibling) and (2) enrolled siblings without SCA but with severe malnutrition (Siblings; Fig. 1). Continuous variables are summarized using means and standard deviations or medians and interquartile ranges for non-normally distributed variables. Categorical variables and prevalence are reported as counts and percentages. Percentages and counts were analyzed using the chi-square or Fisher’s exact test, means were analyzed using the t-test, and medians were analyzed using the Mann-Whitney U test to compare the study and comparison groups. In analyses comparing CwSCA to their Siblings, a clustered analysis was employed to account for individuals from the same household.

Linear regression models were used to compare the final BMI z-scores between groups. Given previous evidence indicating the relationship between weight-for-age z-score and mortality in children with SCA within the same age range in northern Nigeria [12], the same covariates used for the BMI z-score models were used in linear regression models for the final weight-for-age z-scores. In this exploratory study, a 2-sided *P*-value of <.01 indicated a significant result [17]. SPSS version 29.0.1 (IBM, Armonk, NY) and Stata version 18.0 (StataCorp. LLC, College Station, TX) were used for all analyses.

## 3. Results

### 3.1. Sibling enrollment

The study’s main trial results were previously published for the 108 CwSCA who completed the study [13]. Of the 73 siblings without SCA aged 5-12 years old who presented for study screening, 30.1% (n = 22) were severely malnourished (BMI z-score <−3.0). One CwSCA had 2 enrolled siblings, bringing the total number of matched CwSCA with Siblings in the study to 21 (Table 1).

### 3.2. CwSCA with sibling vs CwSCA without sibling

The baseline demographics and anthropometrics of CwSCA with and without Sibling were not significantly different (Table 1). Among CwSCA, 12 (57.1%) of those with an enrolled sibling and 42 (48.3%) of those without an enrolled sibling were randomized to receive hydroxyurea, and this difference was not statistically significant (*p* = .466). The median number of persons in the household, including both adults and children, was 9.0 (IQR: 7.0-14.0) for CwSCA with Sibling and 8.0 (IQR: 6.0-11.0) for CwSCA without Sibling (*P* = .395).

CwSCA with a Sibling had a higher mean change in BMI z-score than CwSCA without Sibling (0.8 vs 0.4, *P* = .003; Table 1). Correspondingly, CwSCA with Sibling were more likely to achieve a BMI z-score of −3.0 or higher at the end of the trial (61.9%, n = 13/21) compared to CwSCA without Sibling (33.3%, n = 29/87, *P* = .016), although this difference did not reach our predefined significance for this exploratory analysis.

We developed a multivariable linear regression model for the final BMI z-scores of the CwSCA. The model included whether the CwSCA had an enrolled Sibling and other covariates (age, sex, and baseline BMI z-score). After adjusting for these variables, we found that having an enrolled Sibling was associated with a higher final BMI z-score in CwSCA ( *β* = 0.369, CI: 0.130-0.607, *p* = .003; Table 2). The baseline BMI z-score was also significant, consistent with the primary trial results [13]. We observed a similar relationship for the final weight-for-age z-score (Supplementary Table 1).

### 3.3. CwSCA with sibling vs their siblings

The baseline demographics of the CwSCA and their Siblings did not differ significantly (Supplementary Table 2). The mean baseline BMI z-scores for the CwSCA (−3.7) were comparable to those of their Siblings (−3.6; *p* = .47; Supplementary Table 2). CwSCA had lower mean baseline height-for-age and weight-for-age z-scores than their Siblings, although this difference did not reach significance at a *P*-value of <.01 (Supplementary Table 2).

The mean change in BMI z-score was 0.8 in the CwSCA and 0.7 in their Siblings, which was not significantly different (*P* = .498, Supplementary Table 2). At the end of the 12-week trial, there was no significant difference in the proportion of CwSCA and Siblings with a BMI z-score of −3.0 or above, with 61.9% (n = 13/21) and 54.5% (n = 12/22), respectively (*P* = .625). In multivariate linear regression models, whether the participant was a CwSCA or a Sibling was not associated with endpoint BMI (*β* = −0.172, CI: −0.424 to 0.081, *P* = .171; Supplementary Table 3). Similarly, there was no significant association with the endpoint weight-for-age z-scores (Supplementary Table 4).

## 4. Discussion

To our knowledge, this is the first study to explore malnutrition treatment outcomes in children with and without SCA in the same household. CwSCA with an enrolled sibling had better treatment outcomes than CwSCA without an enrolled sibling. Our primary analysis found a high rate of sharing the RUTF [13]. Potentially, the additional calories provided to households with enrolled siblings, reduced competition for nutrition in the household and led to the CwSCA consuming more RUTF. A previous study has demonstrated that nutritional interventions can benefit siblings with improved developmental and nutritional outcomes [14]. Our findings support that co-treating siblings with severe acute malnutrition in the household can improve outcomes more effectively for the CwSCA.

We found a high rate of severe acute malnutrition among the siblings screened for participation (30.1%). The severe acute malnutrition prevalence is higher than expected based on general growth norms and exceeds the reported 1.5% prevalence in children aged 6-59 months in Kano, Nigeria [11]. However, the exact prevalence of severe malnutrition in children aged 5-12 years in northern Nigeria is unknown. CwSCA had lower baseline height-for-age and weight-for-age z-scores, indicative of chronic malnutrition, despite having similar baseline BMI z-scores. CwSCA with acute malnutrition exhibited more signs of chronic malnutrition than their siblings, even when living in the same environmental and familial conditions. This chronic malnutrition likely underscores the long-term impact of SCA on growth. Our results align with existing studies that report lower height-for-age z-scores in children with SCA compared to their peers [18,19].

Contrary to our hypothesis, there was no significant difference in treatment response between children with and without SCA. We have preliminary data that over 12 weeks, the prescription of the same amount of calories from RUTF to children with and without SCA was sufficient to achieve a similar change in BMI z-score. Studies in children with HIV have shown similar rates of nutritional recovery in children with and without HIV [20]. In our study, with age-adjusted supplementary calories provided over 12 weeks, most children had improvement in BMI but did not recover from malnutrition, using our study definition of BMI z-score <−3.0. Longer-term follow-up ( >16 weeks) may be needed for children with the most severe baseline nutritional deficits at enrolment [21]. Longer treatment and follow-up could provide a more comprehensive understanding of the sustained impact on nutritional outcomes. We did not assess the nutritional status or caloric needs of other household members outside the 5-12 year age range, which may have influenced outcomes through intra-household resource sharing.

CwSCA with an enrolled sibling showed better nutritional outcomes than CwSCA without an enrolled sibling. In addition, to our knowledge, this is the first study to provide evidence that older malnourished children with and without SCA respond similarly to malnutrition treatment. Therefore, future malnutrition treatment programs for children with SCA should consider screening and treating all children in the household. Treating all malnourished children in a household may improve the outcomes for the index child and help identify specific program components and pathways that lead to positive spillover effects for the index child and the entire household. Treatment outcomes for children who have siblings who are not severely malnourished may also need additional household supplies of RUTF to improve their overall nutritional outcomes.

## Supplementary Material

1

Supplementary material associated with this article can be found, in the online version, at doi:10.1016/j.nutres.2025.10.006.

## Figures and Tables

**Table 1 –:** Baseline characteristics and anthropometric changes for children with sickle cell anemia and severe acute malnutrition (body mass index z-score <−3.0), comparing those with an enrolled sibling (n = 21) to those without an enrolled sibling (n = 87).

Variable	CwSCA with sibling (n = 21)	CwSCA without sibling (n = 87)	*P* value*
Assigned to hydroxyurea group, n (%)	12 (57.1)	42 (48.3)	.466
Age, years, median (IQR)	9.6 (8.8 – 11.4)	10.5 (8.8 – 11.7)	.358
Sex, female, n (%)	13 (61.9)	40 (46.0)	.190
Head of household education, n (%),			.730^#^
*None/Primary/Jr. Secondary*	5 (23.8)	28 (33.3)	
*Sr. Secondary/OND*	13 (61.9)	46 (54.8)	
*University/Professional*	3 (14.3)	10 (11.9)	
Number of persons in the household, median (IQR)	9.0 (7.0 – 14.0)	8.0 (6.0 – 11.0)	.395
Hemoglobin, g/dL, mean (SD)	7.0 (1.0)	7.4 (1.0)	.096
Height, cm, mean (SD)	123.5 (9.6)	123.9 (9.9)	.852
Height-for-age z-score, mean (SD)	−2.10 (0.9)	−2.29 (1.0)	.434
Weight, kg, mean (SD)	18.1 (3.2)	18.7 (3.6)	.493
Baseline Weight-for-age z-score, mean (SD)	−3.51 (0.6)	−3.57 (0.7)	.718
Change in weight-for-age z-score, mean (SD)	0.47 (0.3)	0.24 (0.27)	.001
BMI, kg/m^2^, mean (SD)	11.8 (0.5)	12.1 (0.6)	.075
BMI z-score, mean (SD)	−3.72 (0.4)	−3.65 (0.5)	.526
Change in BMI z-score, mean (SD)	0.80 (0.6)	0.41 (0.5)	.003
BMI z-score ≥−3.0 at 12 weeks, n (%)	13 (61.9)	29 (33.3)	.016

Abbreviations: BMI, body mass index; CwSCA, children with sickle cell anemia; IQR, interquartile range; SD, standard deviation; OND, Ordinary National Diploma.

* Chi-square test for categorical variables, T-test for means, Mann-Whitney U test for medians.

# Fisher’s exact test, *P* values are 2-sided; *α* = 0.05.

Values are expressed as mean ± SD, median (IQR), or count (percentage).

**Table 2 –:** Multivariable linear regression model for 12-week body mass index (BMI) z-score in children with sickle cell anemia and severe acute malnutrition (body mass index z-score <−3.0), comparing those with an enrolled sibling (n = 21) to those without an enrolled sibling (n = 87).

Variable	Beta	95% confidence interval	*P* value
Baseline age	−0.048	−0.096 – 0.000	.049
Sex (female)	−0.144	−0.333 – 0.045	.133
Baseline BMI Z-score	0.664	0.454 – 0.874	<.001
Non-SCA sibling enrolled	0.369	0.130 – 0.607	.003

Abbreviations: BMI, body mass index; SCA, sickle cell anemia; CI, confidence interval.

Values are unstandardized *β* coefficients with 95% confidence intervals and P values from a multivariable linear regression with 12-week body mass index z-score as the dependent variable. Covariates shown are baseline age (years), sex (female), baseline body mass index z-score, and enrollment of a non–SCA sibling. *P* values are 2-sided; *α* < 0.01.

## Abbreviations

AKTH: Aminu Kano Teaching Hospital
BMI: Body mass index
CwSCA: Children with sickle cell anemia
MMSH: Murtala Muhammad Specialist Hospital
RUTF: Ready-to-use therapeutic food
SCA: Sickle cell anemia
