# Supplementary material for: Including malnourished siblings in treatment improves nutritional outcomes for children with sickle cell anemia in Northern Nigeria: Results from a feasibility trial
Source: Nutr Res. Author manuscript; Available in PMC 2026 May 30. (PMC13221909; doi:10.1016/j.nutres.2025.10.006)
Supplement: 1 [file NIHMS2174412-supplement-1.docx]

**SUPPLEMENTARY MATERIAL**

**Including Malnourished Siblings in Treatment Improves Nutritional Outcomes for Children with Sickle Cell Anemia in Northern Nigeria: Results from a Feasibility Trial**

Hassan Adam Murtala, MBBS^1^ Shehu U. Abdullahi, MD, FWACP^2^ Safiya Gambo, MBBS, FWACP,^1^ Halima Kabir, MBBS,^2^ Khadija A. Shamsu, MBBS^2^ Garba Gwarzo, MBBS^2^ Sari A. Acra, MD, MPH^3^ Virginia A. Stallings, MD,^4^ Mark Rodeghier, PhD^5^ Michael R. DeBaun, MD, MPH^6,7^and Lauren J. Klein, MD^3,7^

**Author Affiliations**:

^1^Department of Pediatrics, Murtala Mohammed Specialist Hospital, Kano, Nigeria

^2^Department of Pediatrics, Bayero University/Aminu Kano Teaching Hospital, Kano, Nigeria

^3^Department of Pediatrics, D. Brent Polk Division of Pediatric Gastroenterology, Hepatology, and Nutrition at Monroe Carell Jr. Children's Hospital at Vanderbilt, Nashville, TN, United States

^4^Division of Gastroenterology, Hepatology, and Nutrition, Department of Pediatrics, The Children's Hospital of Philadelphia and University of Pennsylvania, Philadelphia, PA, United States

^5^Rodeghier Consultants, Chicago, IL, United States

^6^Department of Pediatrics, Vanderbilt-Meharry Center of Excellence in Sickle Cell Disease, Vanderbilt University Medical Center, Nashville, TN, United States

^7^Vanderbilt Institute for Global Health, Vanderbilt University Medical Center, Nashville, TN, United States

**Supplementary Table 1**. Multivariable linear regression model for 12-week weight-for-age z-score in children with sickle cell anemia and severe acute malnutrition (body mass index z-score <-3.0), comparing those with an enrolled sibling (n=21) to those without an enrolled sibling (n=87).

| Variable | Beta | 95% Confidence Interval | P Value |
| --- | --- | --- | --- |
| Baseline Age | -0.025 | -0.052 – 0.001 | 0.049 |
| Sex (female) | -0.142 | -0.248 – 0.036 | 0.133 |
| Baseline Weight-for-Age Z-score | 0.975 | 0.896 – 1.054 | <0.001 |
| Non-SCA Sibling enrolled | 0.242 | 0.108 – 0.376 | <0.001 |

Values are unstandardized β coefficients with 95% confidence intervals (CI) and P values from a multivariable linear regression with 12‑week weight‑for‑age z‑score as the dependent variable. Covariates: baseline age (years), sex (female), baseline weight‑for‑age z‑score, and enrollment of a non–SCA sibling. P values are two‑sided; α = 0.05.
Abbreviations: SCA, sickle cell anemia; CI, confidence interval.

**Supplementary Table 2.** Baseline characteristics and anthropometric changes for children with severe acute malnutrition (body mass index z-score <-3.0) compared among children with sickle cell anemia (n=21) to their enrolled siblings without sickle cell anemia (n=22).

| Variable | CwSCA with an enrolled sibling  (n=21) | Enrolled Sibling without SCA  (n=22) | P-Value* |
| --- | --- | --- | --- |
| Age, years, median (IQR) | 9.6 (8.8 – 11.4) | 8.2 (7.0 – 10.7) | 0.145 |
| Sex, female, n (%) | 13 (61.9 | 14 (63.6) | 0.907 |
| Head of household education, n (%), |  | NA | NA |
| *None/Primary/Jr. Secondary* | 5 (23.8) |  |  |
| *Sr. Secondary/OND* | 13 (61.9) |  |  |
| *University/Professional* | 3 (14.3 |  |  |
| Number of persons in the household, median (IQR) | 9.0 (7.0 – 14.0) | NA | NA |
| Hemoglobin, g/dL, mean (SD) | 7.0 (1.0) | 10.8 (1.0) | < 0.001 |
| Height, cm, mean (SD) | 123.5 (9.6) | 123.5 (12.3) | 0.981 |
| Height-for-age z-score, mean (SD) | -2.10 (0.9) | -1.3 (1.1) | 0.010 |
| Weight, kg, mean (SD) | 18.1 (3.2) | 18.1 (4.2) | 0.983 |
| Baseline Weight-for-age z-score, mean (SD) | -3.51 (0.6) | -3.0 (0.8) | 0.022 |
| Change in weight-for-age z-score, mean (SD) | 0.47 (0.3) | 0.40 (0.3) | 0.559 |
| BMI, kg/m^2^, mean (SD) | 11.8 (0.5) | 11.7 (0.6) | 0.560 |
| BMI z-score, mean (SD) | -3.72 (0.4) | -3.6 (0.5) | 0.472 |
| Change in BMI z-score, mean (SD) | 0.80 (0.6) | 0.70 (0.6) | 0.498 |
| BMI z-score >-3.0 at 12 weeks, n (%) | 13 (61.9) | 12 (54.5) | 0.625 |

* Chi-square test for categorical variables, T-test for means, Mann-Whitney U test for medians, # Fisher’s exact test

OND = Ordinary National Diploma; IQR = Interquartile range; SD = standard deviation

Values are mean ± SD, median (IQR), or n (%). P values are two‑sided; α = 0.05.

Abbreviations: BMI, body mass index; SCA, sickle cell anemia; CwSCA, children with sickle cell anemia; IQR, interquartile range; SD, standard deviation; OND, Ordinary National Diploma; NA, not applicable.

**Supplementary Table 3.** Multivariable linear regression model for 12-week body mass index z-score in children with sickle cell anemia and severe acute malnutrition (body mass index z-score <-3.0), comparing children with sickle cell anemia (n=21) to their enrolled siblings without sickle cell anemia (n=22) with clustering by family.

| Variable | Beta | 95% Confidence Interval | P Value |
| --- | --- | --- | --- |
| Baseline Age | -0.101 | -0.202 – -0.001 | 0.048 |
| Sex (female) | -0.167 | -0.201 – 0.535 | 0.355 |
| Baseline BMI z-score | 0.513 | 0.029 – 0.998 | 0.039 |
| Study cohort (sibling) | -0.172 | -0.424 – 0.081 | 0.171 |

Values are unstandardized β coefficients with 95% confidence intervals and P values from a multivariable linear regression with 12‑week body mass index z‑score as the dependent variable. Covariates: baseline age (years), sex (female), baseline body mass index z‑score, and study cohort (sibling). Standard errors were clustered by family. P values are two‑sided; α = 0.05.

Abbreviations: BMI, body mass index; SCA, sickle cell anemia; CI, confidence interval.

**Supplementary Table 4.**  Multivariable linear regression model for 12-week weight-for-age z-score in children with sickle cell anemia and severe acute malnutrition (body mass index z-score <-3.0), comparing children with sickle cell anemia (n=21) to their enrolled siblings without sickle cell anemia (n=22) with clustering by family.

| Variable | Beta | 95% Confidence Interval | P Value |
| --- | --- | --- | --- |
| Baseline Age | -0.035 | -0.877 – 0.018 | 0.188 |
| Sex (female) | -0.033 | -0.224 – 0.289 | 0.793 |
| Baseline weight-for-age z-score | 0.991 | 0.809 – 1.174 | <0.001 |
| Study cohort (sibling) | -0.091 | -0.312 – 0.130 | 0.400 |

Values are unstandardized β coefficients with 95% confidence intervals and P values from a multivariable linear regression with 12‑week weight‑for‑age z‑score as the dependent variable. Covariates: baseline age (years), sex (female), baseline weight‑for‑age z‑score, and study cohort (sibling). Standard errors were clustered by family. P values are two‑sided; α = 0.05.

Abbreviations: SCA, sickle cell anemia; CI, confidence interval.
